# Transfer of disrupted-in-schizophrenia 1 aggregates between neuronal-like cells occurs in tunnelling nanotubes and is promoted by dopamine

**DOI:** 10.1098/rsob.160328

**Published:** 2017-03-08

**Authors:** Seng Zhu, Saïda Abounit, Carsten Korth, Chiara Zurzolo

**Affiliations:** 1 Institut Pasteur, Membrane Traffic and Pathogenesis Unit, 25-28 rue du Docteur Roux, 75724 Paris, France; 2 Department of Neuropathology, Heinrich Heine University Düsseldorf, Düsseldorf, Germany

**Keywords:** schizophrenia, DISC1, aggregates, transfer, dopamine, tunnelling nanotubes

## Abstract

The disrupted-in-schizophrenia 1 (*DISC1*) gene was identified as a genetic risk factor for chronic mental illnesses (CMI) such as schizophrenia, bipolar disorder and severe recurrent depression. Insoluble aggregated DISC1 variants were found in the cingular cortex of sporadic, i.e. non-genetic, CMI patients. This suggests protein pathology as a novel, additional pathogenic mechanism, further corroborated in a recent transgenic rat model presenting DISC1 aggregates. Since the potential role of aggregation of DISC1 in sporadic CMI is unknown, we investigated whether DISC1 undergoes aggregation in cell culture and could spread between neuronal cells in a prion-like manner, as shown for amyloid proteins in neurodegenerative diseases. Co-culture experiments between donor cells forming DISC1 aggregates and acceptor cells showed that 4.5% of acceptor cells contained donor-derived DISC1 aggregates, thus indicating an efficient transfer *in vitro*. DISC1 aggregates were found inside tunnelling nanotubes (TNTs) and transfer was enhanced by increasing TNT formation and notably by dopamine treatment, which also induces DISC1 aggregation. These data indicate that DISC1 aggregates can propagate between cells similarly to prions, thus providing some molecular basis for the role of protein pathology in CMI.

## 1. Introduction

Schizophrenia is a purely clinical diagnosis for a chronic brain disorder that is characterized clinically by positive symptoms such as delusions, thought disorders and hallucinations, as well as negative symptoms such as flattened affect or lack of drive, and cognitive symptoms such as working memory deficits. Anatomical abnormalities such as enlarged ventricles and cellular abnormalities in cortical layering of inter-neurons have been reported in schizophrenia [1], but the pathogenesis of this disease is largely unknown. It is proposed that the disease has a strong neurodevelopmental component [2–4], but that a second hit has to occur in order for the disease to break out and to initiate its chronic course. Genetics has been successful in identifying molecular players related to schizophrenia and other chronic mental illnesses (CMI) [5,6]. The disrupted-in-schizophrenia 1 (*DISC1*) gene was identified in a large Scottish family where the balanced translocation mutation t(1; 11) (q42.1; q14.3) led to C-terminally truncated DISC1 protein, and in an American family where it led to aberrant translation of the C-terminus of the protein [7], which was also significantly associated with schizophrenia and other CMI cases [8]. DISC1 protein is involved in neurite outgrowth and cortical development [9–11], and a wealth of evidence has corroborated *DISC1* as a gene related to behavioural control [3].

Given that proteins found in many sporadic, chronic brain diseases are also found mutated and aggregated in rare familial cases [12], we investigated the solubility status of DISC1 in post-mortem brains of CMI and found it insoluble in 15% of cases with CMI but not healthy controls or patients with neurodegenerative diseases [13,14]. Further studies revealed that DISC1-forming aggresomes were cell-invasive *in vitro* [14] and *in vivo* [15]. Furthermore, a transgenic rat model overexpressing DISC1 and displaying DISC1 aggregates displayed disturbed dopamine homeostasis and behavioural abnormalities [16], supporting the pathogenicity of DISC1 aggregates generated endogenously. These studies established that the DISC1 protein can become misfolded similarly to proteins instrumental in classical neurodegenerative diseases [17], however without causing significant cell death. So far, however, the cell biology mechanism behind DISC1 aggregate formation and function (or dysfunction) has remained unexplored.

Over the past decade, a great number of studies provided evidence for cell-to-cell transmission of various neurodegenerative disease-specific proteins in a prion-like manner [18–20]. The proposed model is that protein aggregates formed in one cell can be passively released by membrane rupture or damage, perhaps accompanying cell death, or be actively released by exocytosis, and in turn be taken up by neighbouring cells [18,19,21]. This newly evolved transmission hypothesis for neurodegenerative diseases not only provides a plausible explanation for the stereotypical spreading patterns of the pathology that have long been observed in multiple diseases, but also offers a fresh perspective on the processes underlying the onset and progression of neurodegeneration [18,19,22]. Of note, the prion-like cell-to-cell transmission is a biological phenomenon of information transfer that does not necessarily have to incur cell death, as yeast prions have clearly demonstrated [23,24]. This is important, since CMI have not been demonstrated to involve neurodegeneration as seen in classical neurodegenerative diseases [25].

Tunnelling nanotubes (TNTs) are membranous F-actin-based conduits connecting remote cells that were first characterized in rat pheochromocytoma (PC12) cells in culture [26]. Subsequent studies confirmed that TNT-like structures were present in different cultured cell types such as epithelial [27], immune [28] and neuronal cells [29–31], with the particularity that they contained actin fibres and did not have any contact with the substratum (bottom of the culture dish or Ibidi). Endosomes, mitochondria, endoplasmic reticulum, calcium and surface proteins were found to pass through TNTs in various cell types [32,33]. Furthermore, TNTs can be hijacked by different pathogens, leading to the spreading of infection [30,34–36]. Interestingly, we have shown that infectious prion particles transferred via TNTs resulted in the transmission of infectivity to the recipient cells [30]. Moreover, amyloid-β (A-β) [37], polyglutamine huntingtin aggregates [29], alpha synuclein [38] and tau [39] were found in TNTs, supporting the hypothesis that they could be a preferential highway for the spreading of proteinaceous aggregates [32,38,39].

In light of these findings, we hypothesized that cell-to-cell spreading of aggregates, so far restricted to neurodegenerative diseases, could apply to DISC1-related CMI, i.e. CMI where DISC1 aggregates are implicated in the pathogenesis. To this aim, by quantitative microscopy we characterized the formation, size and sub-cellular localization of GFP-DISC1 aggregates in neuronal cells. We also show that DISC1 aggregates transfer between neuronal cells in co-culture. This intercellular transfer is not mediated by secretion and uptake, but relies on cell-to-cell contact. Furthermore, only small aggregates transfer between cells and are found inside TNTs; the transfer of DISC1 aggregates is affected by modulation (increase/decrease) of TNT number.

## 2. Results

### 2.1. Characterization of DISC1 aggregate formation in neuronal cells

**Figure 1.**
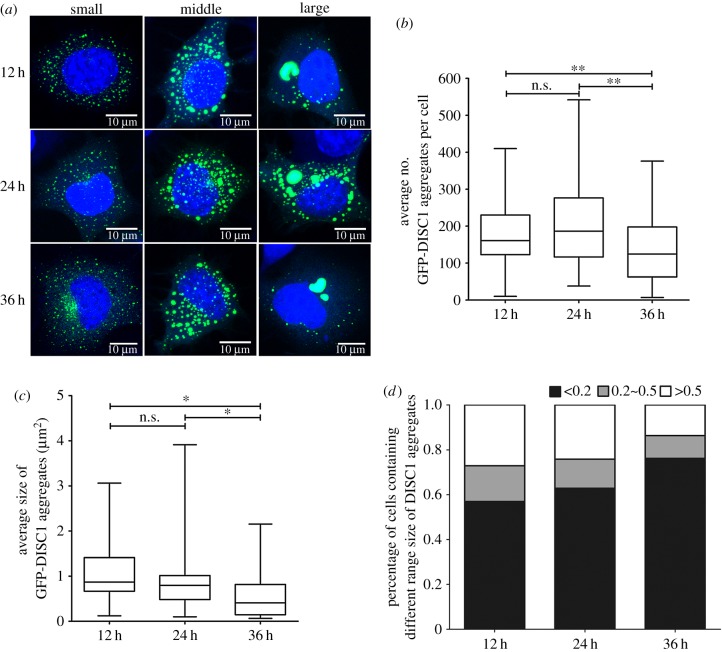
Overexpression of GFP-DISC1 leads to different number and size of aggregates in neuronal cells. (*a*) Representative confocal images of CAD neuronal cells transfected with GFP-DISC1 after 12 h (upper panel), 24 h (middle panel) and 36 h (bottom panel) showing different size of GFP-DISC1 aggregates. Cells contained small (left), medium (middle) and large (right) DISC1 aggregates. Green, GFP-DISC1; blue, cytosolic and nuclear markers (HSC CellMask and DAPI). Scale bars, 10 µm. (*b*) Quantification of the average number of GFP-DISC1 aggregates per cell in (*a*) from three independent experiments (n.s., not significant; **p < 0.01; by two-tailed Mann–Whitney test) showing a decrease in number of DISC1 aggregates 36 h after transfection. (*c*) Quantification of the average size of GFP-DISC1 aggregates per cell in (*a*) from three independent experiments (n.s., not significant; *p < 0.05 by two-tailed Mann–Whitney test) showing a decrease in size of DISC1 aggregates 36 h after transfection. (*d*) Percentage of cells containing small (less than 0.2 µm^2^), medium (0.2–0.5 µm^2^) and large (more than 0.5 µm^2^) aggregates obtained from (*c*).

Recent evidence demonstrated the ability of DISC1 to form insoluble aggregates *in vitro* and *in vivo* [17], however the mechanism of aggregate formation is largely unknown. We first investigated the kinetics of formation of DISC1 aggregates in catecholaminergic murine neuronal-like cells (CAD cells). To do so, we overexpressed GFP-tagged full-length DISC1 protein [14,15] and followed the aggregation process by quantifying the number and size of aggregates at different time points (12 h, 24 h and 36 h) post-transfection. In line with previous reports, we found that GFP-DISC1 formed aggregates in CAD cells at all time points (figure 1*a*). Furthermore, quantification of the number of DISC1 aggregates revealed that while the amount of DISC1 aggregates was similar at 12 h and 24 h post-transfection (on average 161 and 187 DISC1 aggregates per cell at 12 h and 24 h post-transfection, respectively), the number significantly decreased at 36 h after transfection (on average 125 DISC1 aggregates per cell) (figure 1*a*,*b*). Of interest, at this time after transfection, DISC1 aggregates were twice as small as aggregates present in cells at 12 h and 24 h post-transfection (on average 0.41 µm^2^ at 36 h post-transfection compared to 0.87 µm^2^ and 0.80 µm^2^ at 12 h and 24 h post-transfection, respectively) (figure 1*a*,*c*). Consistently, at 12 h and 24 h post-transfection, the percentage of cells containing larger aggregates (more than 0.5 µm^2^) was significantly higher compared to 36 h post-transfection (27.1%, 24.1% and 13.7% of cells containing aggregates more than 0.5 µm^2^ after 12 h, 24 h and 36 h post-transfection) (figure 1*d*). Overall, these data indicate that DISC1 protein forms larger aggregates at relatively short periods after transfection and suggest that DISC1 aggregates can undergo a reduction in size over time, maybe due to proteolysis. In addition, we noticed that small and medium aggregates (less than 0.5 µm^2^) were dispersed in the cytosol while larger aggregates (more than 0.5 µm^2^) were mainly found in the peri-nuclear region (figure 1*a*), compatible with aggresome structures. Time course measurements of lactate dehydrogenase (LDH) release at 12 h post-transfection of GFP-DISC1 showed no change compared to control cells (electronic supplementary material, figure S1). This suggests that cells were viable, thus validating their use as donor cells in our transfer assay (see below).

### 2.2. DISC1 large aggregates form aggresomes in neuronal cells

Based on our previous observations and on the fact that DISC1 was previously shown by us and others to form aggresomes in different neuronal cells [8,17], we next examined whether larger DISC1 aggregates formed 24 h after transfection were localized in aggresome-like structures. By immunostaining CAD cells with bona fide aggresomal markers, we could see most of the larger DISC1 aggregates co-localized with γ-tubulin (electronic supplementary material, figure S2*a*), while most of the small and medium DISC1 aggregates did not. This suggests that DISC1 aggregates may coalesce in large aggregates at the microtubule-organizing centre. We also demonstrated that in cells containing larger DISC1 aggregates, vimentin microfilaments reorganized and engulfed larger DISC1 aggregates (electronic supplementary material, figure S2*b*), in contrast to cells containing small DISC1 aggregates, where the vimentin network was similar to control cells and did not surround the small aggregates (electronic supplementary material, figure S2*b*). Altogether, these results suggest that in CAD cells also, the largest DISC1 aggregates form aggresomes.

We next addressed the sub-cellular localization of small and medium size range DISC1 aggregates by using several organelle markers. We found no co-localization between DISC1 aggregates and early endosomes or lysosomes (electronic supplementary material, figure S3). Only a small number of aggregates co-localized with Vamp3-positive vesicles (13±1.6%, data not shown) (electronic supplementary material, figure S3). These data indicate that the majority of small and medium DISC1 aggregates are not confined in endolysosomal vesicles and might be free in the cytosol.

### 2.3. DISC1 aggregate transfer between neuronal cells is cell-contact dependent

**Figure 2.**
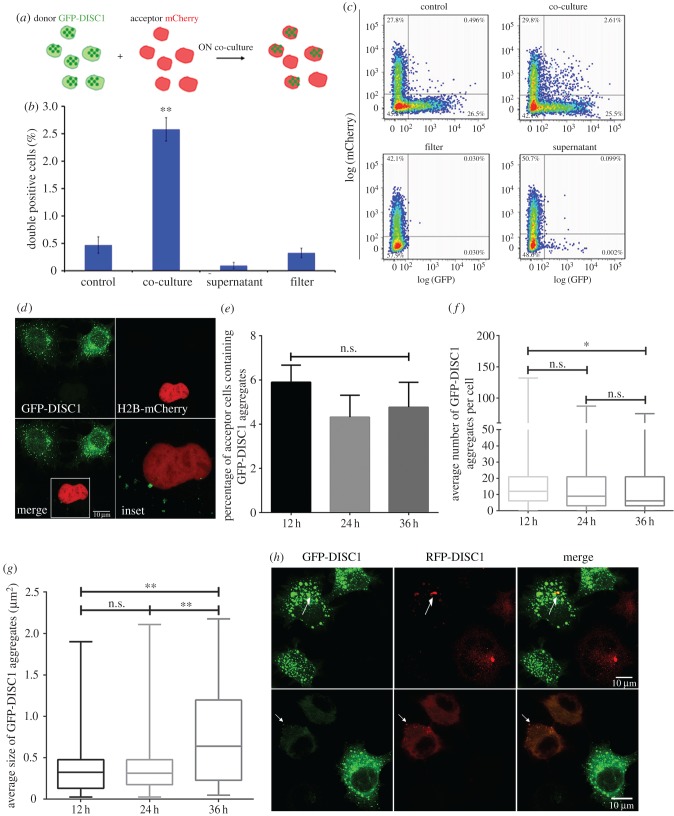
Transfer of DISC1 aggregates between neuronal cells is cell-contact dependent. (*a*) Schematic depicting the co-culture system used to quantify transfer of DISC1 aggregates using flow cytometry. Neuronal donor CAD cells were transfected with GFP-DISC1 for 12 h then co-cultured with acceptor H2B-mCherry transfected cells for 15 h. Cells were then analysed by flow cytometry to quantify the number of double positive cells (i.e. rate of transfer). (*b*) Percentage of double positive cells scored by flow cytometry after three independent co-culture experiments described in (*a*) in control, co-culture, supernatant and filter conditions (see Material and methods). In the co-culture conditions 2.5% of double positive cells were scored, whereas when cell contact was abolished (filter and supernatant conditions) the rate of transfer was drastically decreased. Data show mean ± s.e.m (**p < 0.01 by two-tailed Mann–Whitney test). (*c*) Representative box plots of transfer experiment in (*b*). (*d*) Representative confocal images of donor GFP-DISC1 transfected cells (in green) co-cultured with acceptor H2B-mCherry transfected cells (in red) for 12 h showing several transferred GFP-DISC1 aggregates in acceptor cells (inset). Scale bar, 10 µm (n.s., not significant). (*e*) Quantification of percentage of acceptor cells containing GFP-DISC1 aggregates after co-culture for 12 h, 24 h and 36 h from three independent experiments in (*d*) (n.s., not significant, by two-tailed Mann–Whitney test). Quantification of the average number (*f*) and size (*g*) of GFP-DISC1 aggregates in acceptor cells over the time course from three independent experiments (n.s., not significant; **p < 0.01 by two-tailed Mann–Whitney test) showing that the size of GFP-DISC1 aggregates in acceptor cells increased over time. (*h*) Representative confocal images of GFP-DISC1 and RFP-DISC1 transfected cells co-cultured for 24 h from three experiments. The white arrow points to the co-localized DISC1 aggregates.

We next investigated whether DISC1 aggregates can transfer between CAD cells in culture similarly to prion and prion-like proteins involved in neurodegenerative diseases [21,40,41]. To this end, we set up a co-culture experiment where GFP-DISC1 transfected ‘donor’ cells forming aggregates were co-cultured with H2B-mCherry-transfected ‘acceptor’ cells for 15 h (figure 2*a*). We analysed the rate of transfer by flow cytometry and found 2.5% of double positive cells (i.e. acceptor H2B-mCherry cells containing GFP-DISC1 aggregates) upon overnight co-culture, compared to control conditions (0.5% of double positive cells) (figure 2*b*,*c*). These data suggest that GFP-DISC1 aggregates transferred from one cell population to another in co-culture. To investigate the mechanism of transfer, we examined whether the transfer of DISC1 aggregates relies on secretion. To this aim, we co-cultured donor and acceptor cells separately using a filter that allowed passage of secretory vesicles, but impaired cell-to-cell contact. To directly assess secretion and uptake, we cultured the acceptor cells with the overnight conditioned media of GFP-DISC1 transfected cells. Under both conditions, we observed a large decrease in the rate of intercellular transfer of DISC1 aggregates compared to the co-culture condition (0.50%, 0.32% and 0.09% of double positive cells for mixture control, filter and supernatant conditions, respectively; figure 2*b*,*c*). These data indicate that the intercellular transfer of DISC1 aggregates relies on cell-to-cell contact.

In order to visualize and quantify more precisely the transfer, we combined confocal microscopy with advanced quantitative image analysis using the ICY software (http://icy.bioimageanalysis.org/), which allowed us to identify and characterize the aggregates that were transferred. By carrying out a time course experiment of co-culture post-transfection, we found that around 4.5% of H2B-mCherry acceptor cells contained GFP-DISC1 aggregates and that the transfer rate did not change over time (5.90 ± 0.44% at 12 h, 4.32 ± 0.57% at 24 h and 4.77 ± 0.64% at 36 h) (figure 2*d*,*e*). This result is in line with the flow cytometry analysis. Importantly, we show that transfer is not mediated by the presence of GFP, as in control conditions we observed no transfer of the GFP protein between co-cultures of GFP-vector transfected cells and H2B-mCherry transfected cells (electronic supplementary material, figure S4). To better characterize the transfer of DISC1 aggregates, we measured the number and size of GFP-DISC1 aggregates in acceptor cells. We found that the average number of GFP-DISC1 aggregates in acceptor cells was much less than the number in donor cells (figure 2*d*,*f*). Furthermore, while the size of aggregates in acceptor cells was smaller than the size in donor cells after 12 h and 24 h of co-culture, it increased after 36 h of co-culture (0.39 ± 0.07 µm^2^ post 12 h co-culture, 0.35 ± 0.04% µm^2^ post 24 h co-culture and 0.74 ± 0.07% µm^2^ post 36 h co-culture) (figure 2*d*,*g*). This suggests that after transfer into acceptor cells, DISC1 aggregates might coalesce to form larger aggregates.

It has been shown that in cell where DISC1 aggregates form they are able to co-recruit soluble pools of endogenous DISC1 [42]. Therefore, next we asked whether DISC1 aggregates that have been transferred from donor cells (forming the aggregates) had a similar ability to recruit more DISC1 proteins once arrived in the acceptor cells. To this aim we co-cultured overnight GFP-DISC1 and RFP-DISC1 transfected CADs cells and looked at DISC1 transfer and sub-cellular localization. We found GFP-DISC1 aggregates in the RFP-DISC1 transfected CADs cells, and noticed GFP-DISC1 aggregates co-localized with RFP-DISC1 aggregates. The same event happened in the reverse direction (figure 2*h*), suggesting that DISC1 aggregates can recruit more DISC1 protein after transfer.

### 2.4. DISC1 small aggregates transfer more efficiently than larger aggregates

**Figure 3.**
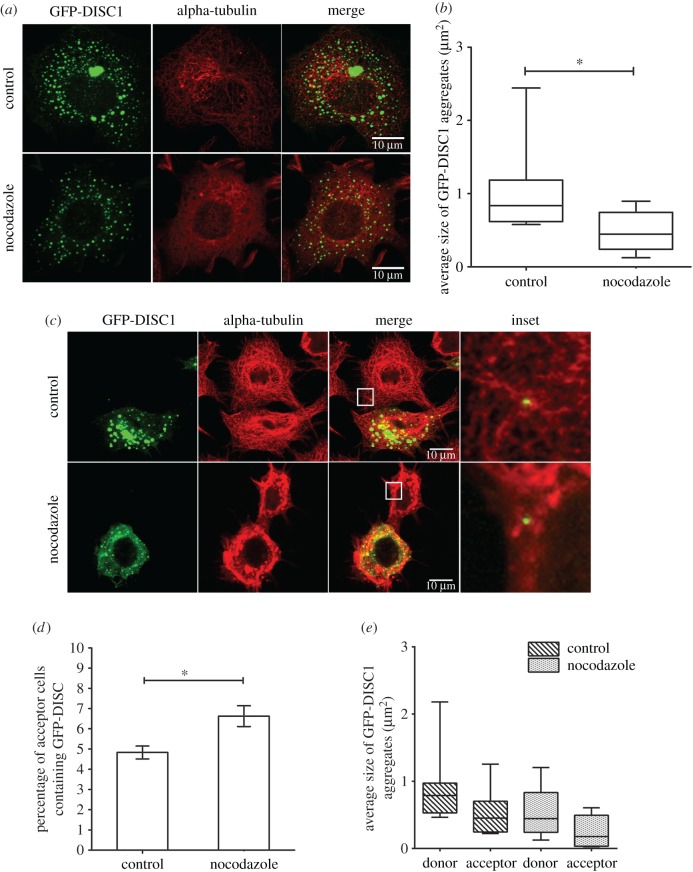
Size of DISC1 aggregates is decreased upon nocodazole treatment. (*a*) Representative confocal images of control and nocodazole treated GFP-DISC1 transfected cells (in green). Immunofluorescence against α-tubulin was used to stain microtubule cytoskeleton (in red), and showed that upon nocodazole treatment the microtubule network is disrupted. Scale bars, 10 µm. (*b*) Quantification of the average number of GFP-DISC1 aggregates in control and nocodazole treated cells in (*a*) from three independent experiments (n.s., not significant; *p < 0.05 by two-tailed Mann–Whitney test) showing an increase of DISC1 size upon nocodazole treatment. (*c*) Representative confocal images of donor GFP-DISC1 transfected cells (in green) co-cultured with untransfected cells for 12 h immunostained for α-tubulin (in red) in control and nocodazole conditions. Transferred GFP-DISC1 aggregates were found in acceptor cells in both conditions. Scale bar, 10 µm. (*d*) Percentage of acceptor cells containing GFP-DISC1 aggregates in (*c*) from three independent experiments (n.s., not significant; *p < 0.05 by Student *t*-test) indicating an increase of DISC1 rate of transfer upon nocodazole treatment. (*e*) Quantification of the average size of GFP-DISC1 aggregates in donor and acceptor cells in (*c*) from three independent experiments showing a decrease in DISC1 aggregates size in both donor and acceptor cells upon nocodazole treatment.

Because aggresome formation requires a microtubule-based cytoskeleton, the microtubule depolymerizing drug nocodazole was shown to cause dispersion of aggresomes [43,44]. We used this approach to investigate the efficiency of transfer of smaller aggregates. As predicted, after nocodazole treatment the size of DISC1 aggregates was significantly reduced (0.83 µm^2^ and 0.45 µm^2^ for control and nocodazole conditions, respectively; figure 3*a*,*b*). In these conditions, more DISC1 aggregates transferred between cells in co-culture compared to controls (4.8% and 6.6% in control and nocodazole conditions, respectively; figure 3*c*,*d*). Consistently, the size of DISC1 aggregates in donor and acceptor cells was significantly reduced upon nocodazole treatment (0.79 µm^2^ and 0.45 µm^2^ for donor cells and 0.45 µm^2^ and 0.18 µm^2^ for acceptor cells in control and nocodazole conditions, respectively; figure 3*e*), indicating that small DISC1 aggregates transferred more efficiently between cells.

### 2.5. DISC1 aggregate transfer is mediated by tunnelling nanotubes

**Figure 4.**
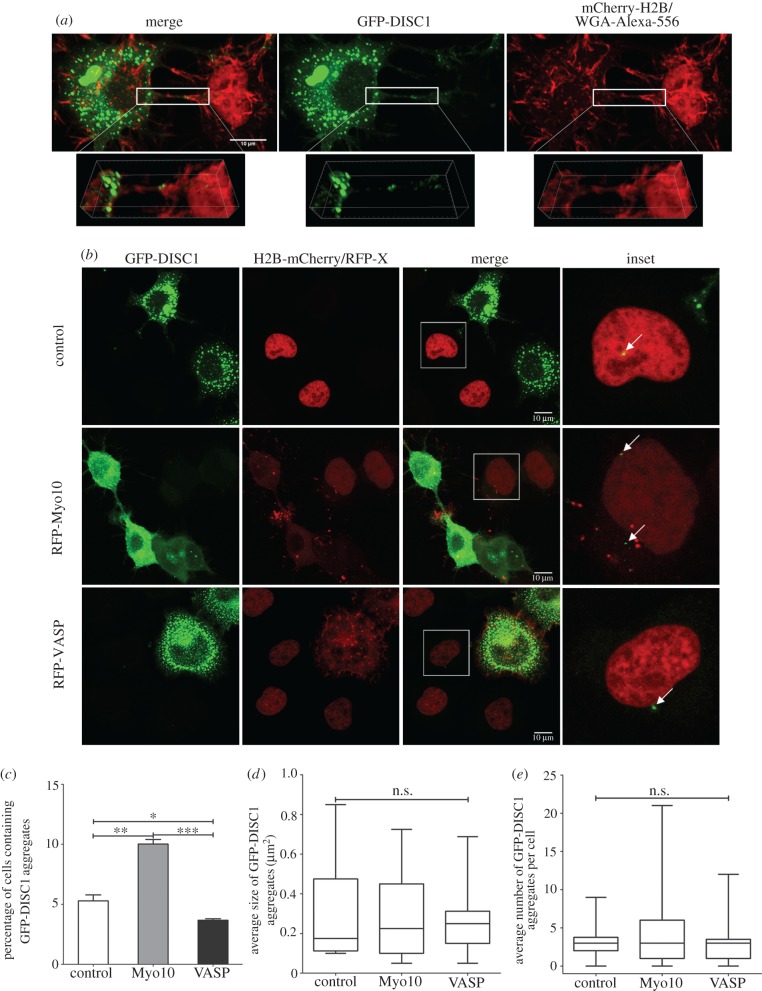
DISC1 aggregates can transfer between neuronal cells through TNTs and the transfer is influenced by TNT formation. (*a*) Representative confocal images of GFP-DISC1 transfected cells (in green) co-cultured with H2B-mCherry transfected cells (nuclei in red). Plasma membrane was stained using WGA-rhodamine (in red). Three-dimensional reconstructions (insets) show the TNTs connecting two cells that contain several DISC1 aggregates (green). Scale bars, 10 µm. (*b*) Representative confocal images of CAD cells co-transfected with GFP-DISC1 and with RFP-Myo10 or RFP-VASP (donor cells) co-cultured with H2B-mCherry transfected cells (acceptor cells). (*c*) Percentage of acceptor cells containing GFP-DISC1 aggregates in (*b*) from three independent experiments. (*p < 0.1; **p < 0.01; ***p < 0.001 by two-tailed Mann–Whitney test). Quantification of the average size (*d*) and number (*e*) of GFP-DISC1 aggregates in acceptor cells in (*b*) from three independent experiments. (n.s., not significant, by two-tailed Mann–Whitney test).

We have previously shown that prion, polyglutamine huntingtin proteins, alpha-synuclein fibrils and tau aggregates use TNTs as an efficient route to transfer between cells [29,30,38–40]. Since transfer of DISC1 aggregates is dependent upon cell-to-cell contact, which is compatible with TNT involvement, we next investigated whether transfer of DISC1 aggregates was mediated by TNTs. By confocal microscopy we found GFP-DISC1 aggregates inside TNTs (figure 4*a*). To test the hypothesis that TNTs can mediate the transfer of DISC1 aggregates, we analysed this transfer in cells overexpressing either Myo10 or VASP, which respectively increase and decrease TNT formation [38]. As shown in figure 4*b*,*c*, the percentage of acceptor cells containing GFP-DISC1 aggregates was increased when TNT formation was induced by Myo10, while the percentage decreased when TNT formation was reduced by VASP (5.28 ± 0.5% in control, 10.02 ± 0.38% in cells co-transfected with Myo10 and 3.68 ± 0.12% in cells co-transfected with VASP). This supports a role for TNTs in the transfer of GFP-DISC1 aggregates between cells. Of note, after co-culturing transfected cells, we also found that the number and size of aggregates in acceptor cells was not changed by either VASP or Myo10 (figure 4*d*,*e*).

### 2.6. Dopamine promotes DISC1 aggregate transfer between neuronal-like cells

**Figure 5.**
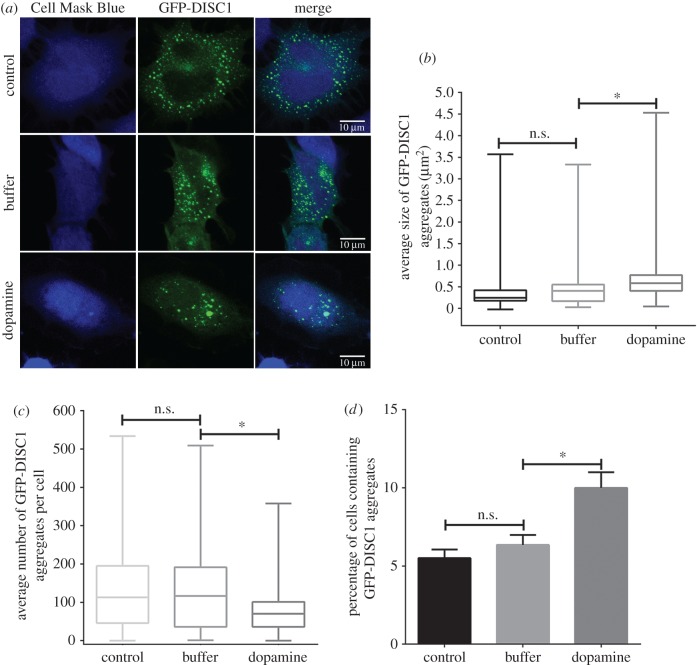
Dopamine increased GFP-DISC1 aggregation and promoted the transfer of aggregates. (*a*) Representative confocal images of GFP-DISC1 transfected cells (in green) and Cell Mask Blue (blue) under control, buffer only and dopamine conditions. Scale bar, 10 µm. Quantification of the average size (*b*) and number (*c*) of GFP-DISC1 aggregates from control, buffer and dopamine treatment GFP-DISC1 transfected cells in (*a*) from three independent experiments. (*d*) The percentage of acceptor cells containing GFP-DISC1 aggregates in co-culture under control, buffer and dopamine conditions. (n.s., not significant; *p < 0.1 by two-tailed Mann–Whitney test).

It has been reported that elevated cytosolic dopamine causes an increase in DISC1 multimerization, insolubility, and complexing with the dopamine transporter [16]; therefore, we wanted to know whether dopamine would affect the formation and transfer of DISC1 aggregates in our system. After cells were incubated with dopamine (100 µM), the size of GFP-DISC1 aggregates increased and the average number of GFP-DISC1 aggregates per cell decreased (figure 5*a*–*c*). This indicates that dopamine does not influence DISC1 expression level, but promotes the formation of DISC1 aggregation. Interestingly, we detected consistently more acceptor cells containing DISC1 aggregates in dopamine-treated cells (5.51 ± 0.55% in normal control condition, 6.35 ± 0.63% in buffer of dopamine condition, 10.00 ± 1.00% in dopamine condition) (figure 5*d*). This supports the physiologically relevant hypothesis that dopamine could promote the formation of DISC1 aggregates and, in turn, their transfer between cells.

## 3. Discussion

Protein aggregates involved in neurodegenerative diseases lead to neuronal dysfunction and neurotoxicity when they accumulate in cells [22,45]. Not all transmissible protein aggregates are cell-toxic, however. For example, yeast prions are transmissible [46] and fulfil physiological functions by increasing their adaptation to starvation [23,24,47–49]. Similarly, DISC1 aggregates have been reported to lead to both loss of function due to the impairment of its binding to biological ligands (e.g. NDEL1) [13] or gain of function by leading to novel interactions [14,16]. Furthermore, large DISC1 aggregates have been shown to have a pathological effect in neurons by disrupting the intracellular transport of key organelle cargoes, such as mitochondria, possibly resulting in a novel DISC1-based mechanism for neuronal pathology [42].

The DISC1 protein consists of large parts with coiled coil regions [50], part of them leading to multimeric interactions or insolubility [51,52]. DISC1 aggregate formation can be accelerated by overexpression [13,14,42], and environmental factors like oxidative stress [16] could induce tertiary structural changes that result in the formation of protein aggregates. There is neither positive nor negative evidence for increased β-sheet structures and/or amyloids in insoluble DISC1 in post-mortem brains [17]; however, endogenous overexpression of DISC1 does not lead to amyloid as measured by thioflavin T staining, contrary to recombinant DISC1 598–785 that clearly forms thioflavin-positive amyloid [16]. Hydrogen peroxide [14,42] and dopamine [16] were found to enhance the formation of DISC1 aggregates, which are recruited into aggresomes around the microtubule-organizing centre, a process that relies on the minus end-directed microtubule motor cytoplasmic dynein.

Recent studies have implicated cell-to-cell transmission of misfolded proteins as a common mechanism for the onset and progression of various neurodegenerative disorders [18]. A prion-like self-propagating mechanism may be applied to a wide range of disease-associated proteins, including Aβ, tau, α-synuclein and polyglutamine huntingtin aggregates [21,22,41]. For these proteins, cell lysates containing aggregates and/or synthetic fibrils assembled from recombinant proteins could act as templates or ‘seeds’ that effectively recruit their soluble counterpart in cultured cells and/or living animals to elongated fibrils [53,54]. For the pathogenesis of chronic mental illnesses such as schizophrenia, protein pathology has not been demonstrated so far, even though phenotypically proteostasis abnormalities can be observed in virtually all chronic brain diseases. Notably, previous investigations in human post-mortem brain [32] and a transgenic rat [16] suggest that protein misassembly of DISC1 could play a role in defining a subset of patients with schizophrenia or other CMI, termed DISC1opathies [17].

In order to test the hypothesis that DISC1 aggregates transfer between neuronal cells, we employed an *in vitro* co-culture system [29,55] in which mouse catecholaminergic neuronal cells (CAD) expressing full-length GFP-DISC1 (forming aggregates) were co-cultured with a distinct population of acceptor cells expressing H2B-mCherry. By flow cytometry, after 24 h of co-culture, we found 2.5% double positive cells, which indicates that GFP-DISC1 aggregates transfer. These results were validated and further characterized by quantitative confocal microscopy, which showed 4.5% average transfer. Importantly, we demonstrated that DISC1 transfer was dependent on cell-to-cell contact and that TNTs, previously shown to mediate the intercellular transfer of amyloidogenic proteins, were involved [29,30,37–39,56,57]. This is the first direct demonstration that GFP-DISC1 aggregates formed in neuronal cells can efficiently transfer to neighbouring cells. Interestingly, our data show that after DISC1 aggregates transfer to other cells they co-localize with DISC1 from the receiving cell in larger aggregates, thus supporting the hypothesis of cell-to-cell transmission of the pathology in a prion-like manner.

When we characterized the time course of formation of GFP-DISC1 aggregates in the CAD cell model, we found that different sizes of DISC1 aggregates were formed over time and that small aggregates did not co-localize with any sub-cellular compartment markers, while they appeared to coalesce in the cytosol in large aggresomes. Consistently, when we disrupted the microtubule network using nocodazole, the formation of DISC1 aggresomes was reduced.

Compared to proteins related to neurodegenerative diseases, the efficiency of transfer of DISC1 aggregates is lower [29,30,56,58,59]. The inefficient transmission of DISC1 aggregates may be due to the low cell invasiveness and limited movement of these aggregates as previously reported [17,42]. By analysing the acceptor cells that received DISC1 aggregates, we found that small aggregates are transferred efficiently between cells. Consistently, when we disrupted aggresome formation with nocodazole, the amount of intercellular transfer of small DISC1 aggregates was increased. Dopamine has been shown to enhance aggregation of the prion protein and of α-synuclein [60,61], while previous data indicated that DISC1 aggregation could be promoted by dopamine [16]. In our hands, after treatment with dopamine the size of GFP-DISC1 aggregates increased, but we detected a consistent increase in the transfer of small DISC1 aggregates. Of interest, dopamine D2 receptor agonist increased polyQ-huntingtin protein aggregation, which was blocked by a dopamine D2 receptor antagonist [62]. Since in schizophrenia baseline occupancy of D2 receptors by dopamine is increased [63] and the CAD cell line expresses D1, D2, D3 and D5 dopamine receptors [64], one possible explanation is that dopamine promotes faster DISC1 aggregation via the D2 receptor. This is consistent with previous evidence showing that elevated cytosolic dopamine causes an increase in DISC1 multimerization, insolubility and complexing with the dopamine transporter [16], and further supports the role of dopamine in the development of CMI.

Several mechanisms could be involved in protein aggregate transmission between cells. In the co-culture system used here, we observed no transmission by supernatant or in the condition of co-culture filter, indicating that the intercellular transfer of DISC1 aggregates is not mediated by secretion and requires cell-to-cell contact. As previously shown for prion and other proteins involved in neurodegenerative diseases [29,30,38,39,56,57], we found that DISC1 aggregates can also transfer between cells through TNTs. This was also supported by the finding that the efficiency of transfer was increased upon induction of TNT formation by Myo10, a positive regulator of TNTs, and reduced by VASP, a negative regulator of TNTs. Our results support the hypothesis that TNTs represent a highway for the intercellular transmission (spreading) of aggregated proteins. It is important to state that our findings, even though they show similarity in the cell-to-cell transmission of protein aggregates seen in neurodegenerative diseases, do not necessarily imply that these aggregates are toxic. In fact, from yeast prions we know that transmissible protein aggregates can fulfil functions, as has also been demonstrated for other cellular systems [65]. How and whether the transmissibility of DISC aggregates is linked to the pathogenesis of schizophrenia remains unclear, and further studies are warranted to decipher the mechanism of DISC1 aggregate formation and its implication in the neuropathology of CMI.

## 4. Material and methods

### 4.1. Cell lines, plasmids and transfection procedures

The mouse catecholaminergic neuronal CAD cell line (mouse catecholaminergic neuronal cell line, Cath.aDifferentiated) was grown in Gibco's OptiMEM supplemented with 10% fetal bovine serum (FBS) and 1% penicillin–streptomycin. GFP-DISC1 and RFP-DISC1 plasmids were from Carsten Korth. RFP-VASP was obtained from Sandrine Etienne-Manneville (Pasteur Institute, Paris, France) and RFP-Myo10 was a gift from Staffan Strömblad (Center for Biosciences, Department of Biosciences and Nutrition, Karolinska Institutet, Stockholm, Sweden). GFP-vector and H2B-mCherry were from AddGene. CAD cells were transiently transfected with Lipofectamine 2000 (Invitrogen) according to the manufacturer's instructions.

### 4.2. Flow cytometry

CAD cells were transfected separately with GFP-DISC1, GFP-vector and H2B-mCherry constructs in 25 cm^2^ flasks as described above.

For co-culture experiments, 12 h after transfection, H2B-mCherry expressing CAD cells were co-cultured with cells expressing either GFP-DISC1 or GFP-vector at a ratio of 1 : 1 in 12-well plates. After 24 h of co-culture, cells were scraped in PBS supplemented with 1% FBS, filtered using a 40 µm nylon cell strainer and fixed in 2% paraformaldehyde (PFA) for flow cytometry analysis (BD Biosciences LSRFortessa cell analyser). Each experiment was performed in triplicate and repeated three times. 10 000 cells were recorded each time.

GFP-DISC1 or GFP-vector-expressing cells were also plated on a 0.4 µm Transwell plate (Costar) placed on top of H2B-mCherry-expressing cells in order to impair cell-to-cell contact. After 24 h of co-culture, filters were removed and H2B-mCherry-expressing cells were analysed by flow cytometry as described above.

In order to test secretion involvement in transfer, CAD cells were transfected separately with GFP-DISC1 and GFP-vector. After 12 h, cells were gently washed with PBS then fresh medium was added for an additional 24 h. This conditioned medium from GFP-DISC1 and GFP-vector CADs was used to culture H2B-mCherry-expressing CADs (transfected the day before). After 24 h of incubation, H2B-mCherry-expressing cells were analysed by flow cytometry as described above.

### 4.3. Immunofluorescence of cells

GFP-DISC1 and GFP-vector transfected CAD cells were plated on Ibidi™ μ-Dishes 35 mm high (Biovalley) for 24 h. Cells were fixed with 4% PFA or cold methanol for vimentin and γ-tubulin experiments. After permeabilization with a blocking solution of 0.01% saponin and 2% BSA, primary antibodies to detect markers of lysosomes (anti-Lamp1, rat), early endosomes (anti-EEA1, rabbit) and ERC (Anti-Vamp3, rabbit, Abcam) were diluted in blocking solution (0.01% saponin and 2% BSA), followed by extensive washing with PBS, secondary antibody addition and washing. Cells were mounted using Aqua-Polymount (Polysciences, Inc.) and images acquired using a Zeiss LSM700 confocal microscope. Co-localization studies were done using an objects-based colocalization method of the ICY software (http://icy.bioimageanalysis.org/).

### 4.4. TNT detection

GFP-DISC1 or GFP-vector–transfected cells were co-cultured for 24 h with cells transfected with H2B-mCherry at a ratio of 1 : 1. Cells were then fixed with fixative solution 1 (2% PFA, 0.05% glutaraldehyde and 0.2 M HEPES in PBS) for 20 min at room temperature, followed by a second 20 min fixation with fixative solution 2 (4% PFA and 0.2 M HEPES in PBS). The cells were gently washed with PBS and labelled with WGA-rhodamine (Sigma; 1 : 300 in PBS) for 20 min at room temperature, washed and sealed with Aqua-Polymount. Image stacks covering the whole cellular volume were acquired using a confocal microscope (Zeiss LSM700). To evaluate the number of TNT-connected cells, manual analysis was performed and only the numbers of GFP-DISC1 or GFP-vector transfected cells, which possessed TNTs, were counted. Each experiment was performed at least in triplicate. Image analyses of raw data, such as Z-projections, were obtained using ICY software [55].

### 4.5. Image processing and quantification

To quantify the percentage of cells containing GFP-DISC1 aggregates and to evaluate the number of TNT-connected cells, a manual analysis was performed as previously described [38]. Experiments were done in triplicate and repeated three times. FACS raw data were analysed using FlowJo software.

To quantify the number of GFP-DISC1 aggregates in CAD cells and their size (expressed as percentage of the cell volume) at the different time points, a computer batch run was performed with ICY software, using aggregates detector plugin. The statistical tests (Tukey's multiple comparisons tests) were performed with Prism software.

### 4.6. Toxicity measurements

Cell death in neuron-like cells was quantified by measuring LDH (LDH plus kit, Roche Diagnostics) release into the culture medium following the manufacturer's instructions. Briefly, CAD cells at 24 h post-transfection with GFP-DISC1 were plated in 96-well plates and kept in culture for 6 h, 16 h, 24 h, 36 h and 48 h. The LDH level corresponding to high (HD) and low neuronal death (LD) as well as background (BK) levels was determined in sister cultures maintained in parallel. To calculate the percentage of cell death in control and experimental conditions the following formula was used after subtraction of BK levels to all measurements: % of cell death = ((experimental value − LD value)/(HD − LD)) × 100.

### 4.7. Dopamine induced DISC1 protein aggregation in CAD cells

CAD cells were cultured with Opti-MEM with 10% FBS and 1% penicillin–streptomycin (Gibco). GFP-DISC1 transfection was performed as above. GFP-DISC1 transfected CAD cells were seeded in Ibidi for 12 h, treated with 100 µM dopamine (with 20 µM ascorbic acid to prevent oxidation) for 24 h and fixed with 4% PFA in PBS. To perform transfer experiments, GFP-DISC1 transfected CAD cells and H2B-mCherry transfected CAD cells were seeded in co-culture for 12 h, treated with 100 µM dopamine (with 20 µM ascorbic acid to prevent oxidation) for 24 h and fixed with 4% PFA in PBS. Cells were stained with Cell Mask Blue and mounted with Aqua-Polymount. Image stacks covering the whole cellular volume were acquired using a confocal microscope (Zeiss LSM700).

## Supplementary Material

Figure S1

## Supplementary Material

Figure S2

## Supplementary Material

Figure S3

## Supplementary Material

Figure S4

## Data accessibility

The datasets supporting this article have been uploaded as part of the electronic supplementary material.
