# Supplementary figures and images for: Transfer of disrupted-in-schizophrenia 1 aggregates between neuronal-like cells occurs in tunnelling nanotubes and is promoted by dopamine
Source: Open Biol. 2017 Mar 8;7(3):160328. doi: 10.1098/rsob.160328 (PMC5376705; doi:10.1098/rsob.160328)

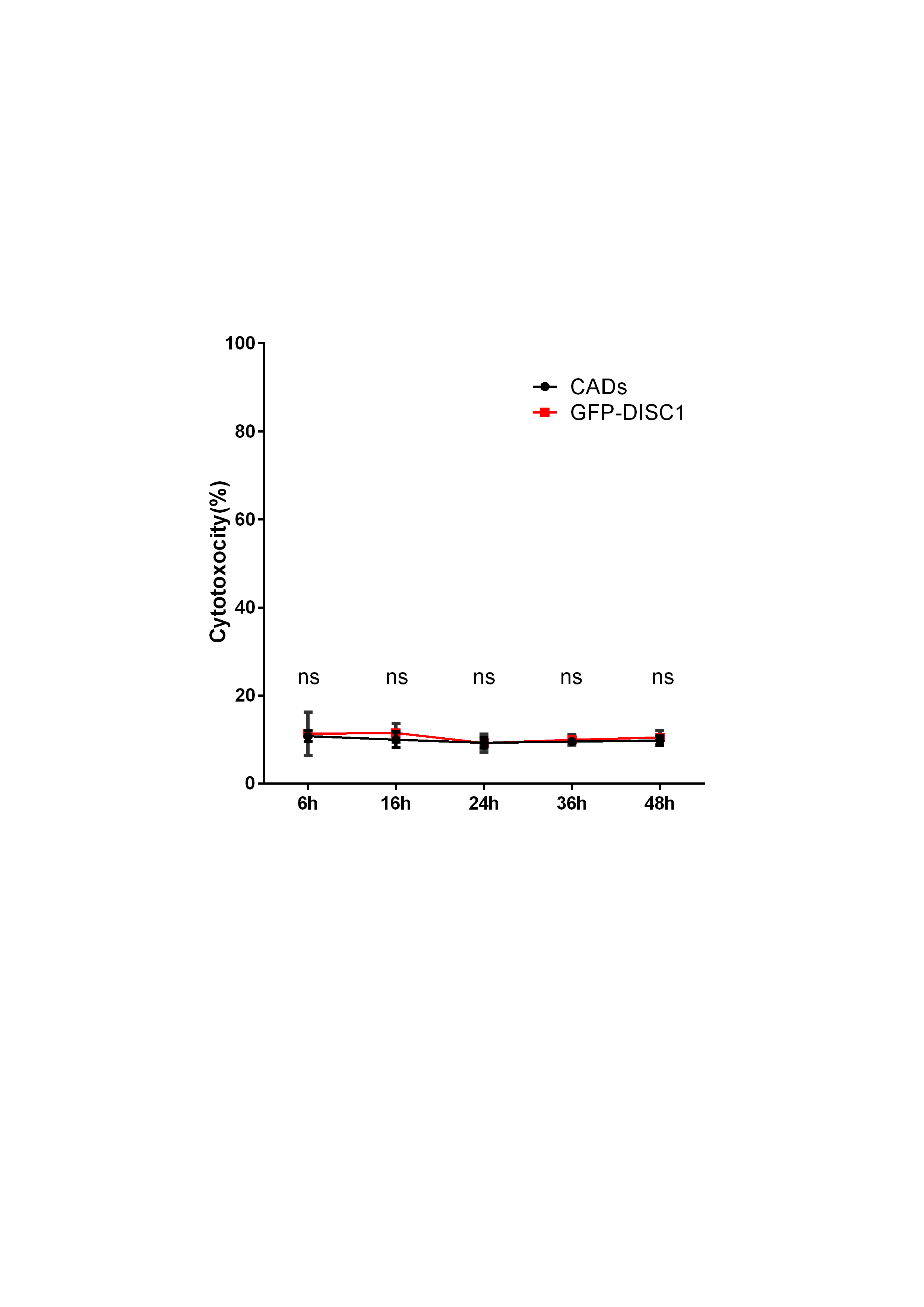

Supplement: Figure S1 [file rsob160328supp1.tif]

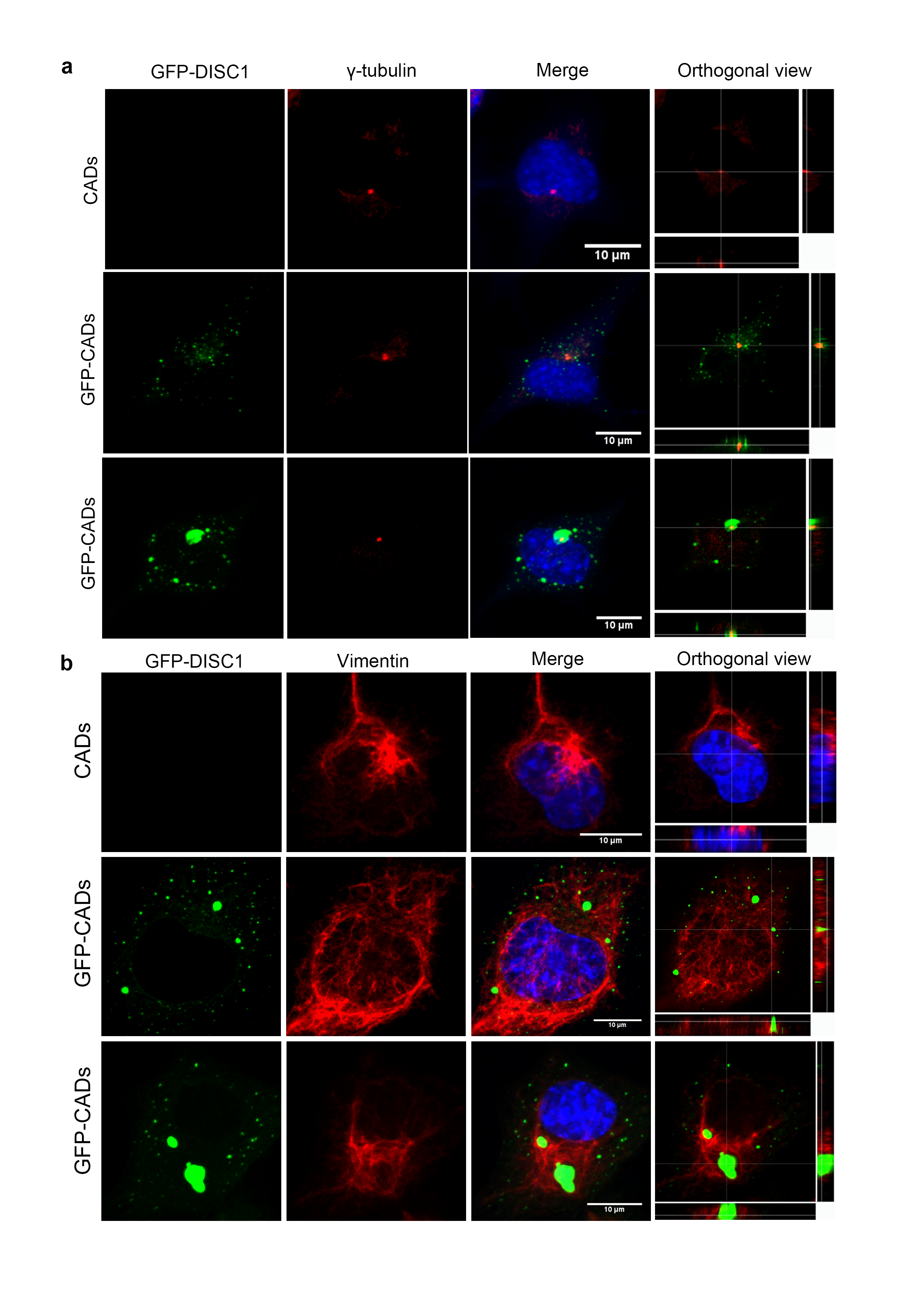

Supplement: Figure S2 [file rsob160328supp2.tif]

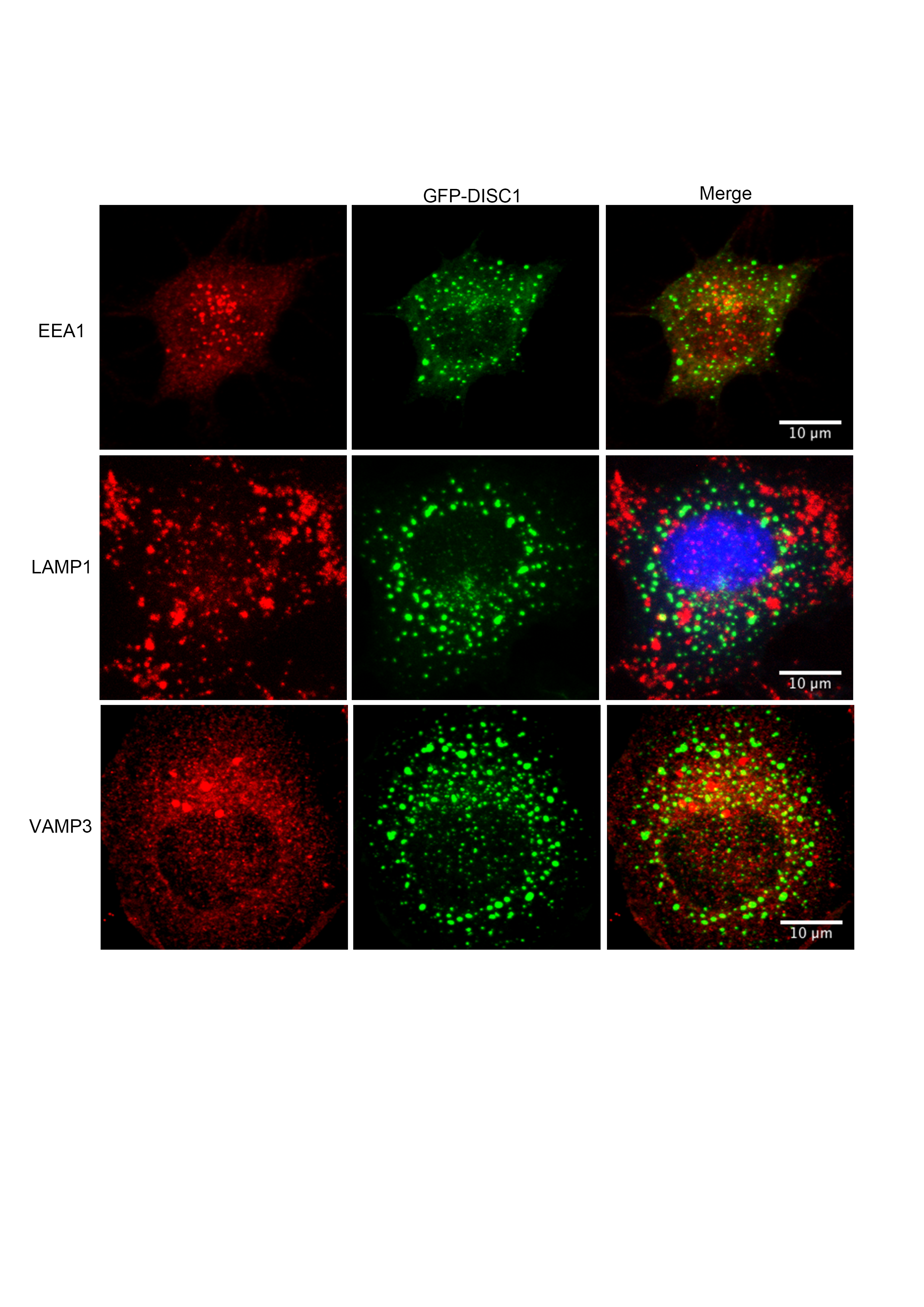

Supplement: Figure S3 [file rsob160328supp3.tif]

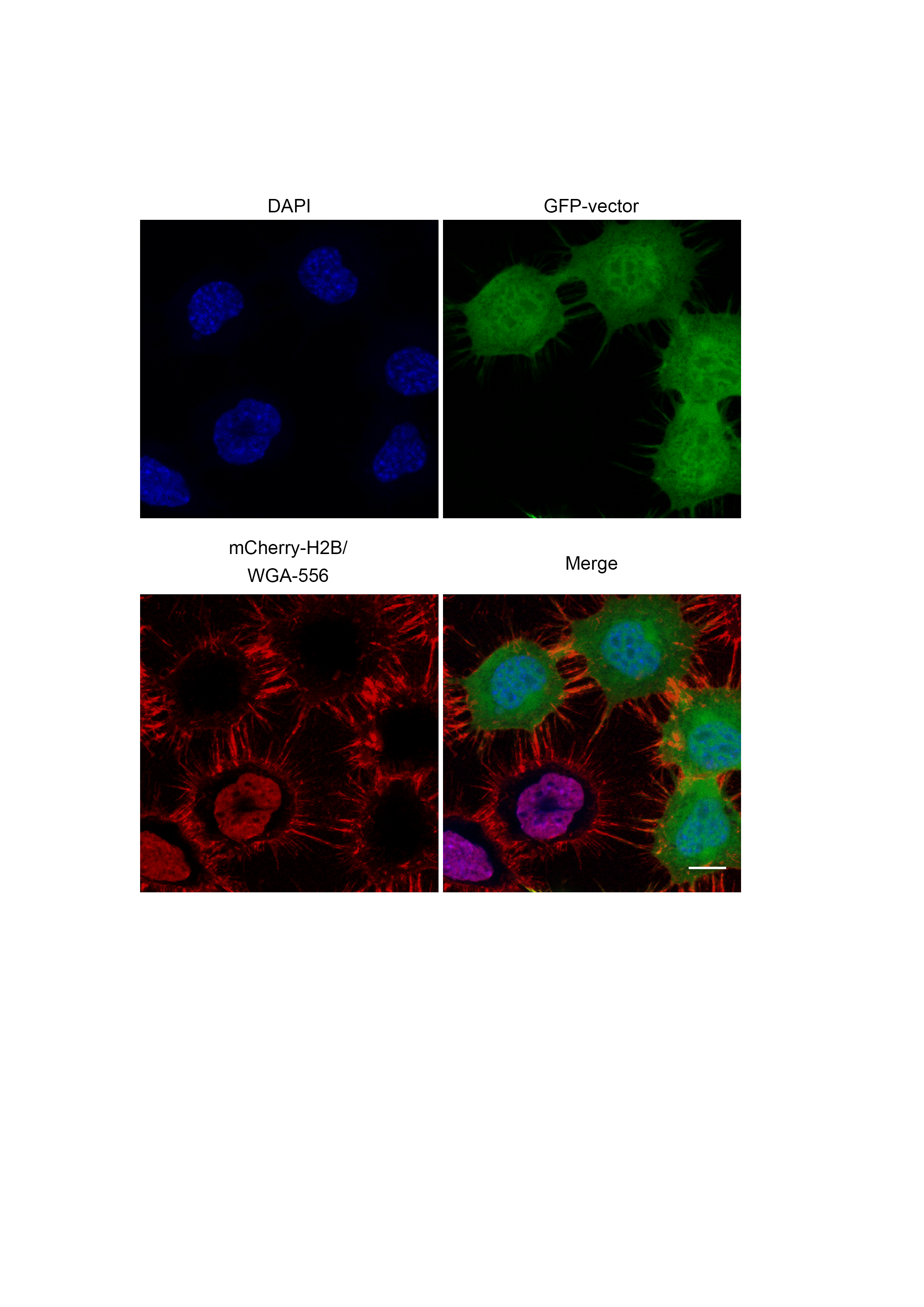

Supplement: Figure S4 [file rsob160328supp4.tif]
